# Potential Factors Predicting Histopathologically Upgrade Discrepancies between Endoscopic Forceps Biopsy of the Colorectal Low-Grade Intraepithelial Neoplasia and Endoscopic Resection Specimens

**DOI:** 10.1155/2022/1915458

**Published:** 2022-06-06

**Authors:** Junbo Hong, Yining Wang, Jiangshan Deng, Miao Qi, Wei Zuo, Yuanzheng Hao, Anjiang Wang, Yi Tu, Shan Xu, Xiaodong Zhou, Xiaojiang Zhou, Guohua Li, Liang Zhu, Xu Shu, Yin Zhu, Nonghua Lv, Youxiang Chen

**Affiliations:** ^1^Department of Gastroenterology, First Affiliated Hospital of Nanchang University, Nanchang, Jiangxi, China; ^2^Department of Respiratory Medicine, First Affiliated Hospital of Nanchang University, Nanchang, Jiangxi 330006, China; ^3^Department of Pathology, First Affiliated Hospital of Nanchang University, Nanchang, Jiangxi 330006, China

## Abstract

**Background:**

It was gradually accepted that endoscopic fragment biopsy (EFB) diagnosis cannot accurately guarantee the absence of higher-grade neoplasms within the lesion of the digestive tract. There are no well-established predictors for histopathologically upgrade discrepancies between EFB diagnosing colorectal low-grade intraepithelial neoplasia (LGIN) and endoscopic resection (ER) specimens.

**Methods:**

A total of 918 colorectal LGINs was histopathologically diagnosed by EFB, including 162 cases with upgrade discrepancy and 756 concordant cases. We compared clinicopathological data of EFB and ER specimens between these two groups. Multivariate analysis was performed to identify predictors for this upgrade histopathology.

**Results:**

The predominant upgrade discrepancy of LGINs diagnosed by EFB was upgrades to high-grade dysplasia (114/918, 12.4%), followed by upgrades to intramucosal carcinoma (33/918, 3.6%), submucosal adenocarcinoma (10/918, 1.1%), and advanced adenocarcinoma (5/918, 0.5%). NSAID history (OR 4.83; 95% CI, 2.27-10.27; *p* < 0.001), insufficient EFB number (OR 2.99; 95% CI, 1.91-4.68; *p* < 0.001), maximum diameter ≥ 1.0 cm (OR 6.18; 95% CI, 1.32-28.99; *p* = 0.021), lobulated shape (OR 2.68; 95% CI, 1.65-4.36; *p* < 0.001), erythema (OR 2.42; 95% CI, 1.50-3.91; *p* < 0.001), erosion (OR 7.12; 95% CI, 3.91-12.94; *p* < 0.001), surface unevenness (OR 2.31; 95% CI, 1.33-4.01; *p* = 0.003), and distal location of the target adenoma (OR 3.29; 95% CI, 1.68-6.41; *p* < 0.001) were associated with the histologically upgrade discrepancies.

**Conclusion:**

NSAID history, insufficient EFB number, adenoma size and location, and abnormal macroscopic patterns are potential predictors for upgrade histopathology of LGINs diagnosed by EFBs. The standardization of EFB number and advanced imaging techniques could minimize the risk of neglecting the potential of this upgrade histopathology.

## 1. Introduction

Among all individuals worldwide in 2018, colorectal cancer (CRC) has become the third most commonly diagnosed cancer for incidence and the second leading cause of cancer death [1]. Early detection and removal of adenomatous polyps (precancerous lesions) can contribute to reduce the occurrence of colorectal cancer [2–3]. As the monoclonal derivatives of a mutated epithelial stem cell, adenomas, the most common type in colorectal polyp, are benign neoplasms that up to 50% of all individuals will develop in their lifetime [4]. Endoscopic resection (ER) for colorectal adenomas is a feasible and safe way with less morbidity, mortality, and costs compared to surgical resection [5]. Endoscopic mucosal resection (EMR) and endoscopic submucosal dissection (ESD) are currently the most used techniques for the resection of large distal colorectal polyps [6–7].

For the intraepithelial neoplasia in the digestive tract, it has been reported that there are some histopathological discrepancies between endoscopic forceps biopsy (EFB) and pathological outcomes of ER specimens, such as superficial esophageal squamous neoplasm, gastric epithelial neoplasia, and colorectal polyps [8–10], whereas few research has been conducted to analyze the factors predicting histopathologically upgrade discrepancies between EFBs of colorectal low-grade intraepithelial neoplasia (LGIN) and ER specimens demonstrating high-grade intraepithelial neoplasia (HGIN) or adenocarcinoma. Therefore, the aim of the present study was to explore these discrepancies and intend to elucidate the potential risk factors and traits contributing to this type of upgrade histopathological diagnosis for the colorectal adenomas with LGIN forceps biopsy, which could suggest us to make a further accurate diagnosis and guide the optimal clinical management for these lesions.

## 2. Patients and Methods

### 2.1. Patients

This retrospective study was based on standard documentation of patients undergoing EMR and ESD in the First Affiliated Hospital of Nanchang University from January 1, 2013, to September 9, 2018.

**Figure 1:**
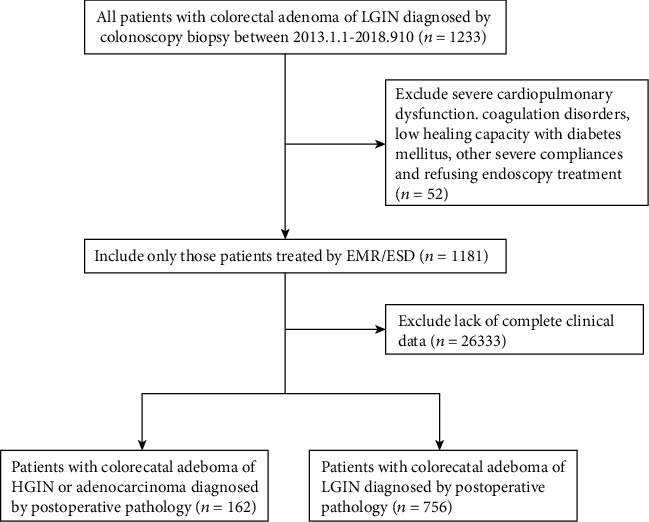
Flow diagram for selecting eligible individuals into histopathologically upgrade and concordant groups.

The participants are patients who were diagnosed with by EFBs demonstrating colorectal adenoma of LGIN. The primary exclusion criteria were endoscopic intolerance with severe cardiopulmonary dysfunction, coagulation disorders, low healing capacity with diabetes mellitus, other severe complications, and refusal to receive endoscopy treatment. Identified by postoperative pathology, patients diagnosed with the colorectal adenomas of HGIN or adenocarcinoma were selected as the case group (histopathologically upgrade group). Patients still diagnosed with colorectal adenoma of LGIN were selected as the control group (histopathologically concordant group). Patients with incomplete data for adenoma characteristics and laboratory examination at baseline were excluded in both two groups. After exclusion, 162 cases and 756 controls were identified (Figure 1).

### 2.2. Data Definition and Collection

We collected patients' characteristics including age, gender, personal medical history of hypertension (HT), and diabetes mellitus (DM), using nonsteroidal anti-inflammatory drug (NSAID), colorectal polyps, CRC, familial adenomatous polyposis (FAP), ulcerative colitis (UC), Crohn's disease (CD), and the number of polyps (single or multiple). We also obtained patients' blood lipid level including triglyceride (TG), total cholesterol (TC), high-density lipoprotein (HDL) and low-density lipoprotein (LDL), and carcinoembryonic antigen (CEA).

We established the principle of selecting a target adenoma from one patient. The adenoma with larger size or abnormal macroscopic characteristics was selected as target if there are multiple adenomas in one individual. We also recorded the clinicopathologic data of target adenomas: site (proximal or distal), adenoma diameter, Yamada classification (I-II or III-IV), the proportion of laterally spreading tumor (LST), macroscopic patterns (lobulation, erythema, erosion, and surface unevenness), standardization for EFB number, EFB histopathology (tubular or tubulovillous or villous), and postoperative pathology (LGIN or HGIN or adenocarcinoma). Based on the consensus, Yamada classification is our reference for assessing the macroscopic type of targeted adenomas [11]. The definition of LST is sessile and flat, ≥10 mm polyps on the basis of the original Kudo classification. Adenomas in the cecum to the transverse colon are defined as “proximal,” and those in the rectum through the splenic flexure are defined as “distal” [12]. The adenoma size was recorded by the maximum diameters of the specimens. The definition of the standardization for the EFB number is at least the integer part of the maximum diameter of the target adenoma. EFB histopathology and postoperative pathology were based on the World Health Organization (WHO) classification [13]. EFB demonstrated that adenomas with 25 to 75% villous features were defined as tubulovillous adenomas, and those with villous architecture ≥ 75% were classified as villous adenomas [14]. HGIN was classified into high-grade dysplasia (HGD) and intramucosal carcinoma. Adenocarcinoma was classified into submucosal adenocarcinoma (submucosa invasion) and advanced adenocarcinoma (muscularis invasion).

### 2.3. Colonoscopy, ER, and Histopathological Reports

Bowel preparation was abstracted on the report forms [15–16]. Conventional white light colonoscopes (CF-H260 series; Olympus, AIZU, Japan/EC-450HL5, EC-450WM5 or EC-590ZW series; Fujinon, Saitama, Japan) were used in the performance of all procedures. Colonoscopy, EMR, and ESD had been randomly allocated to endoscopists who experienced >10,000 colonoscopies (≥1,500 polypectomies) and scrupulously implemented based on the European Society of Gastrointestinal Endoscopy (ESGE) Clinical Guideline [17–19]. The withdrawal times of each colonoscopy were guaranteed at least 6 minutes for adequate inspection [20].

In the cases of EMR, we injected a saline solution containing 0.5% methylene blue and 0.1% epinephrine into the mucosa underneath the basal part of colorectal neoplasms to lift the neoplasm. Then, the neoplasm was snared by the elastic band and resected by a blended electrosurgical current. In the cases of ESD, we injected a saline solution containing 0.5% methylene blue and 0.1% epinephrine in the circumferential edge of the targeted neoplasm into the submucosal space to lift the neoplasm. A circumferential incision was carefully executed by using a needle knife (KD-610L, Olympus, Tokyo, Japan) or a needle knife plus an insulated-tip knife (KD-611L, Olympus, Tokyo, Japan) with subsequent continuous submucosal dissection. For prophylaxis of delayed bleeding, hemostatic forceps (FD-410LR, Olympus) and argon plasma coagulation (APC) were taken to coagulate visible active bleeding vessels in the resected surface of EMR and ESD. For the closure of the wound, a titanium clip and rope were utilized depending on the condition of the wound undergoing EMR and ESD.

The diagnosis of EFBs and ER specimens was assessed and determined by two independent expert gastrointestinal pathologists.

### 2.4. Statistical Analysis

Continuous variables were calculated by using the Student *t*-test and expressed as means ± SD. Age, maximum diameters of adenoma, and blood lipid level were not distributed normally and were expressed as median and interquartile range (IQR) of 25th and 75th percentiles. The Mann–Whitney *U* test was used for statistical analysis of these data. The significance of difference for categorical variables was assessed by chi-square tests or Fisher's exact tests, which were presented as the odds ratio (OR) and 95% confidence interval (CI). Statistical significance was determined at an *α* level of 0.05 by utilizing two-sided tests. Multiple logistic regression was used to identify the significant factors for the phenomenon of this inconsistent pathology.

Post hoc testing was used to measure the predictive significance of the EFB diagnosis and macroscopic type. Multiple variables with *p* < 0.05 in the univariate analysis were selected as potential risk factors in the multivariate forward stepwise logistic regression analysis. All statistical analyses were performed using the Statistical Package for Social Science software suite (version 25.0; SPSS, Inc., Chicago, IL, USA).

## 3. Results

### 3.1. Baseline Characteristics of 918 Patients Suffering LGIN Diagnosed by EFBs

Baseline characteristics of 918 enrolled patients with LGIN diagnosed by EFBs are summarized in Table 1. Among the 918 patients, 584 were male and 332 were female, with a median age of 58 y (IQR: 50-66). 34.7% of patients did not have adequate EFB number. The predominant macroscopic shapes were Yamada III or IV (53.4%). 42 (4.6%) lesions were LSTs. Most lesions (78.5%) were located in the distal large intestine. Lobulated, erythema, erosion, and surface unevenness patterns were observed in 192 (20.9%), 461 (50.2%), 102 (11.1%), and 149 (16.2%) patients, respectively. And 553 cases (60.2%) just suffered single colorectal polyp. Further, the median maximum diameter of the target adenoma is 1.5 cm (IQR: 1, 2). <1.0 cm, 1.0-1.9 cm, 2.0-2.9 cm, and ≥3.0 cm maximum diameters of target adenoma were found in 92 (10%), 487 (53.1%), 233 (25.4%), and 106 (11.5%) patients.

### 3.2. Clinicopathological and Endoscopic Characteristics of Patients in the Histopathologically Upgrade and Concordant Group

Clinicopathologic characteristics of 918 patients are presented in Table 2 between the histopathologically upgrade and concordant groups. The predominant LGIN upgrade discrepancies by EFB diagnosis were upgrades to high-grade dysplasia (114/918, 12.4%), followed by upgrades to intramucosal carcinoma (33/918, 3.6%), submucosal adenocarcinoma (10/918, 1.1%), and advanced adenocarcinoma (5/918, 0.5%). The proportion of patients with HT (29.6 vs. 20.6%, *p* = 0.012), the proportion of patients with NSAID (13.6 vs. 3.8%, *p* < 0.001), insufficient EFB number (63.0 vs. 28.7%, *p* = 0.001), maximum diameter of the target adenoma (2.44 vs. 1.59 cm, *p* < 0.001), lobulated pattern (52.5 vs. 14.2%, *p* < 0.001), erythema (78.4 vs. 44.2%, *p* < 0.001), erosion (42.6 vs. 4.4%, *p* < 0.001), surface unevenness (49.4 vs. 9.1%, *p* < 0.001), and distal location of the target adenoma (88.3 vs. 76.5%, *p* < 0.001) were significantly greater in histopathologically upgrade group. TC (4.33 vs. 4.6 mmol/L, *p* = 0.002) and LDL (2.56 vs. 2.79 mmol/L, *p* = 0.001) were significantly lower in the upgrade group. There was no significant difference in the proportion of patients with DM, colorectal polyps, FAP, and CRC history between the groups. Gender, age, TC, HDL, the proportion of Yamada III+IV classification, LSTs, and multiple colorectal polyps did not differ between two groups.

The significant difference in EFB result (*p* < 0.001) was observed between two groups. Based on post hoc testing, the value of Cramer's V in EFB diagnosis is 0.127 (*p* = 0.001), which indicated a small magnitude of effect size. In the histopathologically upgrade group, the adjusted standardized residual of tubular, tubulovillous, and villous EFB diagnosis were -3.3, +1.9, and +3.2, respectively.

**Figure 2:**
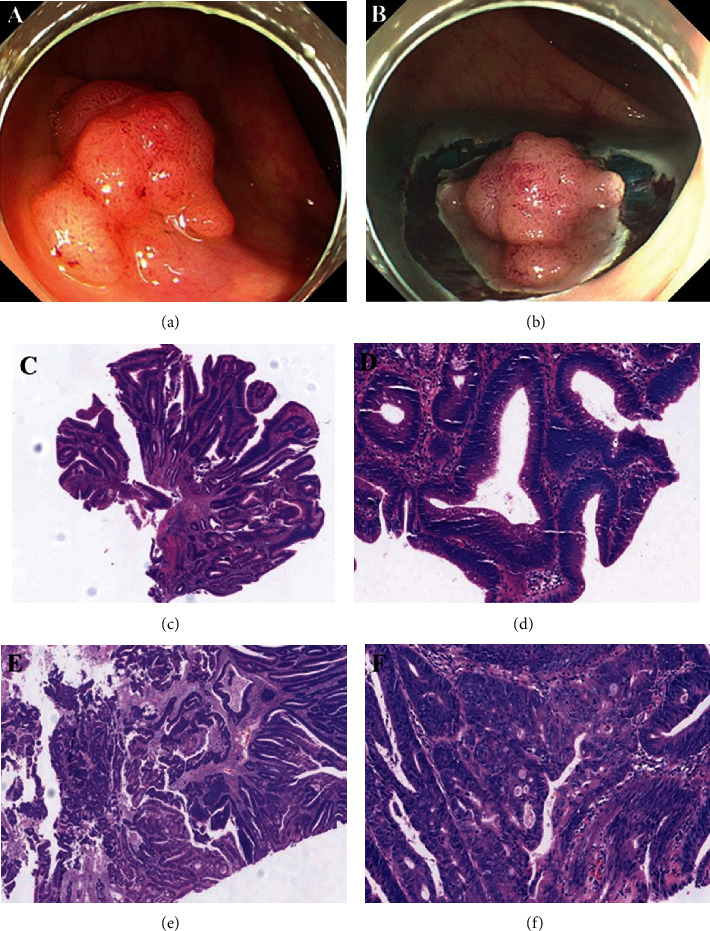
(a) A 20 mm sessile lobulated lesion with erythema and surface unevenness on the rectum. (b) The lesion was excised by endoscopic submucosal dissection. (c) Microscopic features of initial EFB. The biopsy specimen showed mild glandular disarray and stratified epithelial cells with rod-like nuclei. These characteristics were diagnosed with “tubular” low-grade intraepithelial neoplasm (H&E, original magnification ×4). (d) Microscopic features of initial EFB (H&E, original magnification ×20). (e) Microscopic features of ER specimen. The glandular structure on the left side is severely distorted and fused by marked proliferation of disarrayed glands, and epithelial cells showed enlarged and irregular nuclei with nucleoli. These characteristics were consistent with moderately differentiated adenocarcinoma with submucosal invasion (H&E, original magnification ×4). No residual was observed in the tumor margin and base. (f) Microscopic features of ER specimen (H&E, original magnification ×20).

Among the 162 histopathologically upgrade lesions, 15 cases with invasive adenocarcinomas were pathologically confirmed including 5 advanced adenocarcinomas and 10 submucosal adenocarcinomas. Clinicopathological features of 15 patients are shown in Supplement Table 1.Figure 2 shows a typical case with upgrade histopathologically discrepancies between the histological results of EFBs and the ER specimen. This patient was initially diagnosed as LGIN by EFBs and finally confirmed as adenocarcinoma after ER. All lesions among 15 cases are ≥1.0 cm. 93.3% (14/15) of lesions had abnormal macroscopic patterns. 86.7% (13/15) of lesions were distally distributed and had a single piece of EFB.

### 3.3. Predictive Risk Factors for an Initial EFB Diagnosis of LGIN Being Upgraded to a Higher-Grade Histopathologic Diagnosis

Univariate analysis was taken to screen a series of variates including female, age ≥ 60 yrs, HT history, NSAID history, lack of standardization of EFB number, ≥25% villous in EFB diagnosis, maximum diameter ≥ 1.0 cm, lobulated shape, erythema, erosion, surface unevenness, LST, multiple colorectal polyps, and distal location of the target adenoma (Table 3). Multivariate analysis showed that NSAID history (OR 4.83; 95% CI, 2.27-10.27; *p* < 0.001), lack of standardization of EFB number (OR 2.99; 95% CI, 1.91-4.68; *p* < 0.001), ≥1.0 cm maximum diameter of the target adenoma (OR 6.18; 95% CI, 1.32-28.99; *p* = 0.021), lobulated shape (OR 2.68; 95% CI, 1.65-4.36; *p* < 0.001), erythema (OR 2.42; 95% CI, 1.50-3.91; *p* < 0.001), erosion (OR 7.12; 95% CI, 3.91-12.94; *p* < 0.001), surface unevenness (OR 2.31; 95% CI, 1.33-4.01; *p* = 0.003), and distal location of the target adenoma (OR 3.29; 95% CI, 1.68-6.41; *p* < 0.001) were significantly associated with the histologically upgrade discrepancies (Table 3).

## 4. Discussion

Cancers developing from the adenoma towards carcinoma sequence in an indolent pattern may be more likely to be detected and prevented than aggressive cancers progressing rapidly or developing de novo [21–22]. Therefore, precise histopathological diagnoses for superficial colorectal neoplasms are indispensable to select appropriate treatment strategies. EFB samples are usually taken to diagnose the histopathologic grade of targeted colorectal adenomas. In actual clinical practice, some colorectal adenomas diagnosed as LGINs by EFBs were pathologically confirmed as HGINs or adenocarcinomas diagnosed by ER specimens. Recent research has revealed this kind of significantly upgrade discrepancies in histopathological diagnoses between pretreatment EFB and ER specimen for patients with superficial esophageal squamous neoplasms and gastric epithelial neoplasia [8–9]. Some studies also reported histology discrepancies of colorectal polyps but failed to identify potentially predictive risk factors [23–25]. A retrospective study reported histologic discrepancies between pretreatment EFB and EMR specimens of colorectal polyps, but their inclusion criteria are not strict and their sample size is relatively small (290 polyps) [10]. Polyp size > 10 mm was proven as the unique predictor for histology discrepancy and underdiagnosis cases. Our study is to explore potential factors predicting this kind of histopathological upgrade discrepancies for colorectal LGINs diagnosed by EFBs.

An inverse association between the regular NSAID use and the presence of high-risk adenomatous polyps (HRAP) implied a beneficial and chemopreventive effect of NSAID use on the presence of HRAP and recurrence of colorectal adenomas [26–27]. A multicenter, randomized trial confirmed that aspirin (300 mg per day) did not reduce the risk of colorectal adenoma, but it suggests aspirin's chemopreventive activity may possess subtype and location selection [28–30]. NSAID use was a risk factor for upgrade histopathology in our study, which is relatively conflicting to existing data. We considered our result may be reasonable because clinicians are more likely to reduce the number of EFB due to the risk of continuous bleeding in the operation, which increases the possibility of the incidence of upgrade histopathology.

It is meaningful for clinicians to take the fewest number of EFB, while maintaining histopathologic accuracy. We recommend a standardization that EFB number should be obtained at least the integer part of the maximum diameter of a target adenoma, which is valuable experience from the clinical practice in our hospital [31]. The incidence of histopathologic discrepancies is higher in large adenomas due to tumor heterogeneity [32]. We verified ≥1.0 cm could be a significant cut-off value to predict upgrade histopathology in accordance with the previous studies [10–33]. We also analyzed the predictive role of four endoscopic features including lobulated shape, erythema, erosion, and surface unevenness. Flat-type early cancer may be a precursor of advanced cancer in the right colon [34]. And sessile serrated adenomas (SSAs) have been generally considered to associate with microsatellite instability of DNA repair defects. A meta-analysis reported that 8.5% of LSTs contained submucosal invasion (SMI) and a higher risk of SMI was observed in nongranular pseudodepressed LSTs [35]. Hence, we speculated that Yamada classification and LST may predict this upgrade discrepancy, but our result did not show any significance. Further research is required to investigate the predictive role of shape pattern in upgrade histopathology.

There are a series of evidence showing a more distal distribution of colorectal adenomas and advanced adenomas in Asia compared with the U.S., which is identical to our results [36–38]. Additionally, villous adenomas are more likely to be sessile and accompanied with severe atypia or dysplasia. Villous component may imply a risk of rapid growth and malignant tendency so that we speculate a predictive role of EFB diagnosis [39]. However, villous ≥ 25% (tubulovillous and villous) did not reach the significance in our multivariable analysis. Further, we utilized post hoc testing to measure the predictive significance of EFB diagnosis and macroscopic type. For residuals, we set +/-3 as a cut-off value indicating the existence of statistical significance on the basis of general consensus [40–42]. We validated that villous adenoma diagnosed by EFB is significantly related to upgrade discrepancies.

The purpose of our study is to figure out high-risk colorectal adenoma and early cancer in time and achieve early accurate diagnosis. After identifying these predictors, we summarized meaningful strategies to minimize the risk of neglecting these upgrade discrepancies. First, the experience in our institution elucidates that multilayered biopsies should be clamped apically to basally (especially at the nodular, erythematous portion) for high-risk adenomas in order to avoid the neglection of malignant lesions. Second, advanced imaging including narrow-band imaging (NBI), magnifying chromoendoscopy (MCE), confocal laser endomicroscopy (CLE), and blue laser imaging (BLI) is recommended in predicting deep submucosal invasive carcinoma and promoting early diagnosis of T1 CRC dependent on their precise evaluation of the surface pattern, pit pattern, and capillary vessels [43–45]. But high expense and experience variance of interoperator limit the wide spreading of these advanced imaging techniques.

The nature limitations of retrospective studies are inevitable in our study. And the pathologic diagnosis of EFB and ER specimens was assessed by different pathologists which probably leads to interobserver variability [31]. Besides, there is an inevitable selection bias because enrolled individuals were not consecutive due to some exclusion of patients with insufficient clinical data.

## 5. Conclusions

Our results imply that EFB diagnosis of colorectal adenomas with LGINs cannot accurately guarantee the absence of higher-grade neoplasms or carcinoma foci within the lesion. NSAID history, insufficient EFB number, tumor size and location, and abnormal macroscopic patterns are potential predictors for upgrade histopathology of LGINs diagnosed by EFBs. And upgrade histopathology would be far more likely if more risk factors simultaneously existed. Improving the standardization and skills of EFB, advanced imaging techniques could contribute to the accurate detection and diagnosis of colorectal lesions.

## Figures and Tables

**Table 1:** Baseline characteristics of 918 patients with colorectal LGIN diagnosed by EFB.

Baseline characteristics	Value
Median age, *y* (IQR)	58 (50, 66)
Gender, *n* (%)	
Male	586 (63.8)
Female	332 (36.2)
Lack of standardization of EFB number, *n* (%)	
Yes	319 (34.7)
No	599 (65.3)
Shape pattern of the target adenoma, *n* (%)	
Yamada I+II	428 (46.6)
Yamada III+IV	490 (53.4)
LST	42 (4.6)
Location, *n* (%)	
Proximal	197 (21.5)
Distal	721 (78.5)
Lobulated shape, *n* (%)	192 (20.9)
Erythema, *n* (%)	461 (50.2)
Erosion, *n* (%)	102 (11.1)
Surface unevenness, *n* (%)	149 (16.2)
Number of colorectal polyps	
Single	553 (60.2)
Multiple	366 (39.8)
Median largest diameter of the target, cm (IQR)	1.5 (1, 2)
Largest diameter of the target adenoma, *n* (%)	
<1.0 cm	92 (10)
1.0-1.9 cm	487 (53.1)
2.0-2.9 cm	233 (25.4)
≥3.0 cm	106 (11.5)

LGIN: low-grade intraepithelial neoplasia; IQR: interquartile range; EFB: endoscopic fragment biopsy; LST: laterally spreading tumor.

**Table 2:** Baseline characteristics of the concordant group and the upgrade group disease after endoscopic resection.

Characteristics	Upgrade group (*n* = 162)	Concordant group (*n* = 756)	*p* value
Age/y, median (IQR)	59 (48.75, 67)	58 (50, 65)	0.449
Female, *n* (%)	69 (42.6)	263 (34.8)	0.061
Past medical history, *n* (%)			
HT	48 (29.6)	156 (20.6)	0.012
DM	21 (13.0)	71 (9.4)	0.170
NSAID	22 (13.6)	29 (3.8)	<0.001
Colorectal polyps	5 (3.1)	27 (3.6)	0.76
FAP	1 (0.6)	2 (0.3)	0.442
CRC	8 (4.9)	48 (6.3)	0.496
Lack of standardization of EFB number, *n* (%)	102 (63.0)	217 (28.7)	0.001
EFB diagnosis, *n* (%)			<0.001
Tubular	126 (77.8)	663 (87.7)	
Tubulovillous	25 (15.4)	77 (10.2)	
Villous	11 (6.8)	16 (2.1)	
Postoperative pathological diagnosis, *n* (%)			
Tubular adenoma	—	712 (77.6)	
Tubulovillous adenoma	—	34 (3.7)	
Villous adenoma	—	10 (1.1)	
HGD	114 (12.4)	—	
Intramucosal carcinoma	33 (3.6)	—	
Submucosal adenocarcinoma	10 (1.1)	—	
Advanced adenocarcinoma	5 (0.5)		
Maximum diameter of the target adenoma/cm, median (IQR)	2.44 (1.5, 3)	1.59 (1, 2)	<0.001
Lobulated pattern, *n* (%)	85 (52.5)	107 (14.2)	<0.001
Erythema, *n* (%)	127 (78.4)	334 (44.2)	<0.001
Erosion, *n* (%)	69 (42.6)	33 (4.4)	<0.001
Surface unevenness, *n* (%)	80 (49.4)	69 (9.1)	<0.001
Blood biochemistry, median (IQR)			
TG, mmol/L	1.14 (0.80, 1.63)	1.27 (0.89, 1.84)	0.025
TC, mmol/L	4.33 (3.73, 5.08)	4.6 (4.03, 5.24)	0.002
HDL, mmol/L	1.27 (1.03, 1.49)	1.25 (1.05, 1.52)	0.742
LDL, mmol/L	2.56 (2.04, 3.10)	2.79 (2.25, 3.30)	0.001
Shape pattern, *n* (%)			0.547
Yamada I+II	79 (48.8)	349 (46.2)	
Yamada III+IV	83 (51.2)	407 (53.8)	
LST, *n* (%)	5 (3.1)	37 (4.9)	0.318
Multiple colorectal polyps	73 (45.1)	293 (38.8)	0.137
Distal location of the target adenoma, *n* (%)	143 (88.3)	578 (76.5)	<0.001

HT: hypertension; DM: diabetes mellitus; NSAID: nonsteroidal anti-inflammatory drug; FAP: familial adenomatous polyposis; CRC: colorectal cancer; HGD: high-grade dysplasia; TG: triglyceride; TC: total cholesterol; HDL: high-density lipoprotein; LDL: low-density lipoprotein.

**Table 3:** Multivariate analyses for predictive factors demonstrating histopathologically upgrade discrepancies between pretreatment EFB and endoscopic resection specimens.

Characteristics	Univariate analysis		Multivariable analysis	
	OR (95% CI)	*p* value	OR (95% CI)	*p* value
Female	1.39 (0.98,1.97)	0.061		0.054
Age ≥ 60	1.18 (0.84, 1.66)	0.337		0.256
Past medical history				
NSAID	3.94 (2.20, 7.06)	<0.001	4.83 (2.27, 10.27)	<0.001
Colorectal polyps	0.86 (0.33,2.27)	0.760		
FAP	2.34 (0.21,25.98)	0.488		
CRC	0.77 (0.36,1.65)	0.497		
Lack of standardization of EFB number	4.22 (2.96, 6.03)	<0.001	2.99 (1.91, 4.68)	<0.001
≥25% villous in EFB diagnosis	2.04 (1.33, 3.13)	0.001		0.845
Maximum diameter ≥ 1.0 cm	10.81 (2.64, 44.36)	0.001	6.18 (1.32, 28.99)	0.021
Lobulated shape	6.70 (4.63, 9.69)	<0.001	2.68 (1.65, 4.36)	<0.001
Erythema	4.59 (3.07, 6.85)	<0.001	2.42 (1.50, 3.91)	<0.001
Erosion	16.26 (10.18, 25.95)	<0.001	7.12 (3.91, 12.94)	<0.001
Surface unevenness	9.71 (6.54, 14.42)	<0.001	2.31 (1.33, 4.01)	0.003
LST	0.62 (0.24, 1.60)	0.322		
Multiple colorectal polyps	1.30 (0.92, 1.83)	0.138		
Distal location of the target adenoma	2.32 (1.40, 3.85)	<0.001	3.29 (1.68, 6.41)	<0.001

## Data Availability

The data used to support the findings of this study are available from the corresponding author upon request.

## Abbreviations

EFB: Endoscopic fragment biopsy
LGIN: Low-grade intraepithelial neoplasia
ER: Endoscopic resection
CRC: Colorectal cancer
EMR: Endoscopic mucosal resection
ESD: Endoscopic submucosal dissection
HGIN: High-grade intraepithelial neoplasia
HT: Hypertension
DM: Diabetes mellitus
NSAID: Nonsteroidal anti-inflammatory drug
FAP: Familial adenomatous polyposis
CD: Crohn's disease
TG: Triglyceride
TC: Total cholesterol
HDL: High-density lipoprotein
LDL: Low-density lipoprotein
CEA: Carcinoembryonic antigen
OR: Odds ratio
CI: Confidence interval
HRAP: High-risk adenomatous polyps
LST: Laterally spreading tumors
SMI: Submucosal invasion
SSA: Sessile serrated adenomas
NBI: Narrow-band imaging
MCE: Magnifying chromoendoscopy
BLI: Blue laser imaging
CT: Computed tomography
EUS: Endoscopic ultrasound.

## Supplementary Materials

Supplementary Materials
Supplement Table 1: baseline characteristics of 15 lesions upgraded from LGIN in the EFB specimen to adenocarcinoma in the endoscopic resection specimen. M: male; F: female; HT: hypertension; DM: diabetes mellitus; NSAID: nonsteroidal anti-inflammatory drug; CRC: colorectal cancer; Lob: lobulated shape; Ery: erythema; Ero: erosion; SU: surface unevenness; LST: laterally spreading tumor; T: tubular; V: villous; TV: tubulovillous; TG: triglyceride; H-M: high-moderate; M-P: moderate-poor; NR: not reported; M: moderate; MM: muscularis mucosa; SM: submucosa; TC: total cholesterol; HDL: high-density lipoprotein; LDL: low-density lipoprotein; CEA: carcinoembryonic antigen.
