# Supplementary material for: Potential Factors Predicting Histopathologically Upgrade Discrepancies between Endoscopic Forceps Biopsy of the Colorectal Low-Grade Intraepithelial Neoplasia and Endoscopic Resection Specimens
Source: Biomed Res Int. 2022 Jun 6;2022:1915458. doi: 10.1155/2022/1915458 (PMC9192244; doi:10.1155/2022/1915458)
Supplement: Supplementary Materials — Supplement Table 1: baseline characteristics of 15 lesions upgraded from LGIN in the EFB specimen to adenocarcinoma in the endoscopic resection specimen. M: male; F: female; HT: hypertension; DM: diabetes mellitus; NSAID: nonsteroidal anti-inflammatory drug; CRC: colorectal cancer; Lob: lobulated shape; Ery: erythema; Ero: erosion; SU: surface unevenness; LST: laterally spreading tumor; T: tubular; V: villous; TV: tubulovillous; TG: triglyceride; H-M: high-moderate; M-P: moderate-poor; NR: not reported; M: moderate; MM: muscularis mucosa; SM: submucosa; TC: total cholesterol; HDL: high-density lipoprotein; LDL: low-density lipoprotein; CEA: carcinoembryonic antigen. [file 1915458.f1.pdf]

Patient	Sex	Age (yrs)	Location	Past medical history	Endoscopic image	Macroscopic shape	Maximum tumor size (cm)	Number of colorectal polyps	EFB diagnosis	Number of endoscopic biopsies	Pathology	Differentiation	Invasive depth	Positive residual tumor margin and base	TG, mmol/L	TC, mmol/L	HDL, mmol/L	LDL, mmol/L	CEA, ng/ml
1	F	56	Distal	-	Lob, Ery, Ero, SU	LST	2.5	1	T	1	Advanced adenocarcinoma	M-P	SM	Yes	0.58	3.99	1.3	2.26	0.77
2	M	61	Distal	-	Ery	III	1.8	3	T	1	Advanced adenocarcinoma	M	Serosa	No	1.18	5.52	1.22	3.84	5.74
3	M	60	Distal	DM	Lob, Ery, Ero, SU	III	2	1	T	1	Advanced adenocarcinoma	M	SM	Yes	1.3	4.8	1.62	3.05	1.9
4	F	77	Distal	-	Ery, Ero	II	2	3	T	1	Advanced adenocarcinoma	M	SM	Yes	1.4	4.17	0.78	2.65	2.53
5	F	78	Distal	HT	Ery	III	2	1	TV	1	Advanced adenocarcinoma	H-M	SM	Yes	1.5	4.74	0.59	2.42	3.07
6	F	66	Proximal	-	Lob, Ery, Ero, SU	I	2	2	T	1	Submucosal adenocarcinoma	M	SM	No	1.14	4.85	1.85	2.56	3.74
7	F	52	Distal	HT, NASID	Lob, Ery, Ero, SU	II	4	1	T	1	Submucosal adenocarcinoma	M	SM	No	3.16	4.11	0.89	2.17	2.32
8	M	68	Distal	HT	Lob, Ery, SU	II	2	1	T	1	Submucosal adenocarcinoma	M	SM	No	0.73	6.8	1.27	5.06	1.6
9	F	62	Distal	HT	Lob, Ery, Ero, SU	III	3.5	1	T	1	Submucosal adenocarcinoma	M	SM	No	2.29	2.77	0.43	1	1.41
10	M	61	Distal	-	Ery, Ero, SU	IV	2	3	T	1	Submucosal adenocarcinoma	M	SM	No	1.34	5.04	1.49	2.99	3.06

11	F	66	Distal	-	-	II	2.5	2	T	1	Submucosal adenocarcinoma	M	SM	No	2.45	4.12	0.97	2.41	1.83
12	M	77	Distal	-	Ery, Ero	IV	2.5	3	T	1	Submucosal adenocarcinoma	M	SM	No	0.8	3.71	1.41	2	1.12
13	M	66	Proximal	HT, CRC	Lob, Ery, Ero	III	1.5	3	T	1	Submucosal adenocarcinoma	M	SM	No	0.96	4.36	0.98	0.59	1.63
14	M	71	Distal	DM, NSAID	Ery	I	2	3	V	1	Submucosal adenocarcinoma	M	SM	No	0.97	4.56	0.59	2.42	3.99
15	F	46	Distal	-	Ery, SU	III	2	1	T	1	Submucosal adenocarcinoma	M	MM	Yes	0.69	3.71	0.59	2.42	0.99

Supplement Table 1. Baseline characteristics of 15 lesions upgraded from LGIN in the EFB specimen to adenocarcinoma in the endoscopic resection specimen. M, male; F, female; HT, hypertension; DM, diabetes mellitus; NSAID, nonsteroidal anti-inflammatory drug; CRC, colorectal cancer; Lob, lobulated shape; Ery, erythema; Ero, erosion; SU, surface unevenness; LST, laterally spreading tumor; T, tubular; V, villous; TV, tubulovillous; TG, triglyceride; H-M, high-moderate; M-P, moderate-poor; NR, not reported; M, moderate; MM, muscularis mucosa; SM, submucosa; TC, total cholesterol; HDL, high-density lipoprotein; LDL, low-density lipoprotein; CEA, carcino-embryonic antigen
